# Social support and physical literacy in young and middle-aged patients with hypertension: the mediating effects of sense of coherence and self-efficacy

**DOI:** 10.1186/s12888-024-05935-5

**Published:** 2024-07-08

**Authors:** Guiyue Ma, Chunqing Zhou, Zhihao Han, Tingyu Mu, Xiaoqin Ma

**Affiliations:** 1 School of Nursing, Zhejiang Chinese Medical University, Hangzhou, 310053 China; 2 School of Nursing, Anhui University of Chinese Medicine, Hefei, 230012 China; 3 School of Nursing, Anhui Medical University, Hefei, 230032 China

**Keywords:** Social support, Physical literacy, Sense of coherence, Self-efficacy, Patients with hypertension

## Abstract

**Background:**

Despite the growing recognition of the importance of social support and physical literacy in managing hypertension among young and middle-aged patients, there is a lack of research exploring the mediating effects of sense of coherence and self-efficacy in this relationship. This study aims to bridge this gap by investigating the interplay between social support, physical literacy, sense of coherence, and self-efficacy, thus contributing to a deeper understanding of effective interventions for hypertension management.

**Methods:**

A cross-sectional study was conducted using convenience sampling to survey 280 young and middle-aged patients diagnosed with hypertension from five community settings in Zhejiang and Anhui provinces between January and February 2024. Measurement instruments included the General Information Questionnaire, Physical Literacy Scale for Young and Middle-aged Patients with Hypertension, Sense of Coherence Scale 13, General self-efficacy Scale, and Perception Social Support Scale. Data analysis was performed using SPSS 27.0 and AMOS 28.0, with reporting following the STROBE checklist.

**Results:**

A total of 270 valid questionnaires were collected. The total score of physical literacy for young and middle-aged patients with hypertension ranged from 18 to 90, with a mean score of 62.30 ± 13.92, indicating a moderate level. There was a positive correlation between the physical literacy score and the scores of social support (*r* = 0.557, *P*<0.01), sense of coherence (*r* = 0.392, *P*<0.01), and self-efficacy (*r* = 0.466, *P*<0.01) among young and middle-aged patients with hypertension. Furthermore, social support was found to have multiple mediating effects through sense of coherence and self-efficacy on physical literacy.

**Conclusion:**

This study sheds light on the interconnectedness of social support, physical literacy, sense of coherence, and self-efficacy among young and middle-aged patients with hypertension. The findings underscore the importance of considering these factors holistically in hypertension management strategies.

## Introduction

In recent years, the number of people with hypertension has continued to rise, with a growing prevalence among younger populations [1]. Currently, there are about 245 million patients with hypertension in China, 67.5% of whom are young and middle-aged individuals [2]. Over the past thirty years, the number of young and middle-aged patients with hypertension has sharply increased, with a growth rate of up to 95.9% [1]. This increase has led to a significant rise in the burden of hypertension-related diseases, particularly cardiovascular diseases. However, awareness, treatment, and control rates of hypertension among young and middle-aged patients are significantly lower than those among older patients and urgently need improvement [3]. Young and middle-aged patients have a high long-term risk of cardiovascular disease and are a focal point for prevention efforts. Strengthening effective control and management of hypertension in these patients can help prevent and reduce cardiovascular diseases, achieving the “Healthy China 2030” strategic objectives.

Risk factors for young and middle-aged patients with hypertension include a lack of exercise, stress, obesity, high salt intake, and smoking or excessive drinking [4]. Advocating a healthy lifestyle is crucial in managing hypertension among this group. The focus should be on controllable factors to regulate high blood pressure. Physical activity is recognized as an effective non-drug treatment, demonstrating significant effects in preventing cardiovascular diseases [5]. However, with the advancement of technology, people’s physical activity has decreased, leading to the widespread phenomenon of sedentary lifestyles. Studies have revealed that the physical activity status of patients with hypertension in China is inadequate, especially among young and middle-aged patients, whose activity levels fall significantly below the global average [6]. Therefore, increasing the physical activity level of young and middle-aged patients with hypertension stands as an important and urgent real-world problem to address.

Physical Literacy (PL) is described as ‘the motivation, confidence, physical competence, and knowledge and understanding required to engage in physical activities’ [7]. Physical literacy helps young and middle-aged patients with hypertension maintain purposeful physical activity. Improving physical literacy can significantly boost physical activity, enhance cardiopulmonary function and muscle strength, and help maintain a healthy weight and body fat ratio [8]. It also plays a crucial role in regulating blood pressure and lipid levels, thereby preventing the onset and progression of cardiovascular diseases [9]. Moreover, good physical literacy benefits mental health by reducing anxiety and depression, boosting self-confidence and enthusiasm, and improving work efficiency and quality of life [10]. Despite these benefits, research on physical literacy in young and middle-aged patients with hypertension remains insufficient. Therefore, actively promoting the enhancement of physical literacy in this population is of great importance.

The perspective of health origin, as an emerging concept, advocates for maintaining and enhancing individuals’ health status through proactive lifestyles and healthy behaviors. Central to this perspective are the sense of coherence (SOC) and generalized resistant resources, which highlight the importance of health resources and the process of health promotion [11]. Sense of coherence, as an individual’s overall cognition and grasp of life, helps patients maintain a positive mindset when facing challenges such as chronic diseases like hypertension [12]. Social support, a critical component of external resources, provides the necessary conditions and impetus for individuals to tackle challenges and sustain healthy behaviors [13]. Self-efficacy, the core of internal resources, reflects individuals’ beliefs and expectations about their own capabilities and serves as a key internal force driving changes in health behaviors [14]. Specifically, social support plays a crucial role in improving the physical literacy of young and middle-aged patients with hypertension by providing emotional, informational, and practical assistance. This support includes help from family, friends, and medical institutions. Research indicates that support from family and friends encourages patients to persist in physical activities, while medical institutions enhance physical literacy by offering necessary opportunities, resources, and support [15]. Engaging in social networks, communicating with others, and sharing experiences in managing hypertension not only provide motivation and positive feedback but also promote patients’ engagement in beneficial physical activities.

Sense of coherence arises when individuals perceive a consistency between their actions and values. It encompasses three components: comprehensibility, manageability, and meaningfulness [16]. When individuals experience alignment between their behaviors and value systems, they gain increased confidence and motivation to face challenges and difficulties, thereby enhancing their physical and mental well-being. Those with a high level of sense of coherence are better at understanding the nature of difficulties, possess the knowledge and skills to solve problems, and can adapt to stressors and challenges more effectively [17]. Additionally, the impact of social support plays a crucial role in enhancing sense of coherence. Strong social support networks provide individuals with the emotional and practical resources needed to perceive their lives as more manageable and meaningful. This support fosters a sense of belonging and security, further strengthening their ability to cope with life’s challenges [18]. Enhancing sense of coherence through social support contributes to more active participation in physical activities, strengthens individuals’ motivation and confidence in these activities, and deepens their health understanding and perception through experiential learning. Consequently, this improvement in physical literacy leads to better overall health outcomes.

Self-efficacy, on the other hand, refers to an individual’s confidence in their ability to perform a specific behavior, which is directly related to whether patients can proactively engage in physical activity [19]. It involves an individual’s evaluation of and beliefs about their own capabilities, closely linked to their behavior, emotional state, and cognitive status. When engaged in physical activities, individuals with sufficient confidence can better cope with various situations. Studies have shown that people with high self-efficacy are more likely to fully participate in physical activities, whereas those with low self-efficacy may avoid physical activities to escape potential embarrassment or disappointment [20]. Furthermore, the impact of social support on self-efficacy cannot be understated. Supportive relationships provide encouragement and positive reinforcement, which boost individuals’ confidence in their abilities to engage in and maintain physical activity. Individuals with adequate knowledge and understanding are better able to recognize and appreciate the benefits of physical activity, thereby becoming more motivated to participate and improve their physical literacy [21]. In this way, social support not only enhances self-efficacy but also promotes sustained engagement in health-enhancing behaviors.

The relationship between sense of coherence and self-efficacy is well-supported by theoretical and empirical literature. Antonovsky’s sense of coherence theory suggests that individuals with a strong sense of coherence are more likely to feel confident in their ability to manage stressors, which aligns with the concept of self-efficacy [22]. Empirical studies have demonstrated that a strong sense of coherence is positively correlated with higher self-efficacy, as individuals who perceive their lives as comprehensible, manageable, and meaningful are more likely to believe in their capacity to exert control over their health behaviors [23]. For instance, a study by Van Wilder et al. [24]. found that sense of coherence was significantly associated with self-efficacy in managing chronic illness, highlighting the interplay between these constructs in promoting health and well-being. Thus, enhancing both sense of coherence and self-efficacy through social support can lead to improved health outcomes and increased engagement in physical activity.

Understanding the intricate interplay among social support, sense of coherence, self-efficacy, and physical literacy is crucial for enhancing the physical literacy of young and middle-aged patients with hypertension. While social support, sense of coherence, and self-efficacy are individually recognized as influential factors in fostering physical literacy, the theoretical foundation supporting the mediation hypothesis requires further elucidation. Health origin theory has elucidated the interconnections among these factors. Social support can strengthen patients’ sense of coherence and enhance their psychological resilience when facing disease-related challenges [25]. When young and middle-aged patients with hypertension feel that their health behaviors are acknowledged and supported, their motivation and confidence to persist with these activities are boosted, thereby fostering an improvement in physical literacy [26]. Therefore, this study proposes the following hypotheses: Hypothesis 1: The level of social support among patients with hypertension is positively correlated with their physical literacy, indicating that higher levels of social support are associated with higher levels of physical literacy. Hypothesis 2: Sense of coherence mediates the relationship between social support and physical literacy among patients with hypertension, indicating that social support influences physical literacy through sense of coherence. Hypothesis 3: Self-efficacy mediates the relationship between social support and physical literacy among patients with hypertension, indicating that social support influences physical literacy through self-efficacy. Hypothesis 4: Sense of coherence and self-efficacy play a serial mediating role between social support and physical literacy among patients with hypertension, indicating that social support influences physical literacy through a sequential process involving both sense of coherence and self-efficacy.

The purpose of this study is to explore the intricate pathways through which social support influences physical literacy, considering the mediating roles of sense of coherence and self-efficacy. By examining these relationships, we seek to provide theoretical support for the development of more precise and effective intervention strategies for physical literacy among young and middle-aged patients with hypertension, thereby promoting the enhancement of patients’ physical literacy.

## Methods

### Study design and participants

This cross-sectional study was conducted in Zhejiang and Anhui provinces, China, between January and February 2024. A convenience sampling method was used to recruit young and middle-aged patients diagnosed with hypertension from five community settings. The inclusion criteria were: (1) aged between 18 and 59 years; (2) diagnosed with hypertension according to the 2020 National Guidelines for the Prevention and Treatment of Hypertension in Primary Health Care [27]: systolic blood pressure (SBP) ≥ 140 mmHg and/or diastolic blood pressure (DBP) ≥ 90 mmHg; 3) able to understand and complete the questionnaire. Exclusion criteria were: (1) severe cardiovascular or cerebrovascular diseases; (2) cognitive or communication impairments. Of the 280 participants invited to participate, 270 valid questionnaires were collected.

### Measures

#### General information questionnaire

Designed by the researchers to collect demographic data including gender, age, marital status, education level, occupation, average monthly household income per capita, family location, living situation, type of medical insurance, duration of illness, and presence of comorbidities.

#### Physical literacy scale for young and middle-aged patients with hypertension

Our research team developed the initial draft of the item pool through literature review, interviews, and focused group meetings. We then conducted two rounds of expert consultations to refine the item pool, resulting in a preliminary draft of the physical literacy scale for young and middle-aged patients with hypertension. This draft included 4 dimensions and a total of 23 items. Following a pre-survey to screen the items, an 18-item scale was finalized and its reliability and validity were verified using a questionnaire survey. The final version of the scale consists of 4 dimensions and 18 items: cognition (5 items), emotion (3 items), physical competence (5 items), and behavior (5 items). The scale uses a Likert 5-point scoring method, with scores ranging from 1 to 5, representing “strongly disagree” to “strongly agree,” respectively. Rigorous reliability and validity testing demonstrated excellent psychometric properties, with a Cronbach’s alpha coefficient of 0.94, indicating good reliability and validity.

#### Sense of coherence scale 13 (SOC-13)

This study utilized the short version of the Sense of Coherence Scale (SOC-13) translated by Bao Leiping et al. [28]. , consisting of three dimensions and a total of 13 items: manageability, comprehensibility, and meaningfulness. The questionnaire employed a 7-point Likert scale, ranging from 1 (“Never”) to 7 (“Very often”). Scores ranged from 13 to 91, with items 1, 2, 3, 8, and 13 reverse-scored. Scores were categorized as low (13–63), moderate (64–79), and high (80–91). The total score reflected the level of an individual’s sense of coherence, with higher scores indicating better SOC levels. The Cronbach’s α value was 0.89.

#### General self-efficacy scale

The General Self-efficacy Scale, revised by Professor Wang Caikang from South China Normal University [29], consists of 10 items measured on a 4-point Likert scale ranging from 1 (“completely disagree”) to 4 (“completely agree”). Total scores range from 10 to 40, with higher scores indicating greater self-efficacy. The scale demonstrated good reliability, with a Cronbach’s α coefficient of 0.87.

#### Perception social support scale

The Perceived Social Support Scale, adapted and translated by Jiang Qianjin based on Zimet’s original version [30], includes 12 items across three dimensions: family support (items 3, 4, 8, 11), friend support (items 6, 7, 9, 12), and other support (items 1, 2, 5, 10). It uses a 7-point scoring system, with scores ranging from 12 to 84. Total scores are categorized into low support (12–36), moderate support (37–60), and high support (61–84), indicating higher levels of perceived social support. The scale exhibited good reliability, with a Cronbach’s α coefficient of 0.84.

### Data collection

A survey team, comprising researchers and two trained master’s students, conducted the data collection process. The team obtained consent from the responsible persons at the research sites and distributed the questionnaires to eligible patients. Patients were briefed on the anonymous and confidential nature of the survey, its purpose, and significance, and signed informed consent forms. The surveyors collected completed questionnaires on-site and ensured their integrity.

### Statistical analysis

Data were uniformly coded, double-entered, and subjected to statistical analysis using SPSS 27.0 software. A significance level of α = 0.05 was adopted, with *P* < 0.05 considered statistically significant. Descriptive statistics, Kolmogorov-Smirnov and Levene tests, independent sample t-tests, Mann-Whitney U tests, Kruskal-Wallis H tests, and Pearson correlation analysis were conducted. Structural equation modeling (SEM) was used to analyze the hypothetical models. SEM, a comprehensive method for testing relationships among variables, was conducted using the Analysis of Moment Structures (AMOS, version 28.0). The reporting followed the STROBE checklist.

## Results

### General characteristics of young and middle-aged patients with hypertension

A total of 270 valid questionnaires were collected, yielding a response rate of 96.4%. The demographic characteristics of the participants are summarized in Table 1. Among the participants, the proportion of males (54.8%) was higher than that of females (45.2%). The largest age group was those aged 51~59 years old (44.8%). Furthermore, the majority of participants were married, accounting for 92.2%.

**Table 1:** General characteristics of young and middle-aged patients with hypertension (*n* = 270)

Items		*N*	%	The score for physical literacy (points, x̅±s)
Gender	Male	148	54.8	60.12 ± 14.89
	Female	122	45.2	64.95 ± 12.19
Age (years)	18~40	65	24.1	63.35 ± 14.65
	41~50	84	31.1	63.89 ± 12.40
	51~59	121	44.8	60.64 ± 14.44
Marital status	Unmarried	21	7.8	63.62 ± 9.45
	Married	249	92.2	62.19 ± 14.24
Educational level	Primary school and below	65	24.1	59.66 ± 15.86
	Junior high school	71	26.3	59.49 ± 14.99
	High school	81	30.0	63.49 ± 13.53
	Bachelor degree or above	53	19.6	67.49 ± 7.75
Occupation	Unemployed	62	23.0	62.08 ± 15.01
	On-the-job	208	77.0	62.37 ± 13.62
Average monthly household income per capita	<1000	34	12.6	62.38 ± 11.47
	1000~3000	86	31.9	62.35 ± 14.83
	3001~5000	78	28.9	63.23 ± 11.78
	>5000	72	26.7	61.21 ± 16.05
Family location	City	201	74.4	61.83 ± 14.42
	Rural	69	25.6	63.68 ± 12.35
Living situation	Live with one’s family	251	93.0	63.30 ± 13.46
	Living alone	19	7.0	49.11 ± 13.58
Type of medical insurance	At one’s own expense	13	4.8	57.46 ± 12.84
	New rural cooperative medical system	61	22.6	59.46 ± 15.45
	Basic medical insurance for urban workers	96	35.6	63.57 ± 13.90
	Basic medical insurance for urban residents	86	31.9	63.84 ± 12.26
	Commercial medical insurance	7	2.6	59.14 ± 20.25
	Others	7	2.6	63.00 ± 13.06
Duration of illness (years)	1~10	172	63.7	59.05 ± 14.53
	11~20	90	33.3	67.34 ± 10.83
	21~30	8	3.0	75.63 ± 3.46
Presence of other comorbidities	None	170	63.0	62.79 ± 13.79
	Have	100	37.0	61.48 ± 14.18

### Physical literacy status of young and middle-aged patients with hypertension

The results showed that the physical literacy scores among the 270 young and middle-aged patients with hypertension varied from 18 to 90, with a mean score of 62.30 ± 13.92, indicating a moderate level of physical literacy. Detailed scores for each dimension are provided in Table 2.

**Table 2:** Scores of physical literacy for young and middle-aged patients with hypertension (points, *n* = 270)

Item	Score range	Score (x̅±s)	Mean score of items
Total score	18~90	62.30 ± 13.92	3.46 ± 0.77
Cognition	5~25	19.29 ± 5.24	3.86 ± 1.05
Emotion	3~15	11.00 ± 3.06	3.67 ± 1.02
Physical competence	5~25	15.85 ± 4.52	3.17 ± 0.90
Behavior	5~25	16.17 ± 4.21	3.23 ± 0.84

### Social support, sense of coherence, and self-efficacy status in young and middle-aged patients with hypertension

The results showed that the total score for social support among young and middle-aged patients with hypertension ranged from 12 to 84, with a mean score of 55.14 ± 14.32, indicating a moderate level. The total score for sense of coherence ranged from 13 to 91, with a mean score of 59.27 ± 13.60, indicating a low level. The total score for self-efficacy ranged from 10 to 40, with a mean score of 24.55 ± 6.24, indicating a moderate level. See Table 3 for details.

**Table 3:** Scores of social support, sense of coherence, and self-efficacy in young and middle-aged patients with hypertension (scores, *n* = 270)

Item		Score range	Score (x̅±s)	Mean score of items
Social support	Total score	12~84	55.14 ± 14.32	4.60 ± 1.19
	Family	4~28	18.42 ± 5.29	4.61 ± 1.32
	Friend	4~28	18.69 ± 5.02	4.67 ± 1.26
	Others	4~28	18.03 ± 5.05	4.51 ± 1.26
Sense of coherence	Total score	13~91	59.27 ± 13.60	4.56 ± 1.05
	Comprehensibility	5~35	21.62 ± 5.77	4.32 ± 1.15
	Manageability	4~28	18.52 ± 5.40	4.63 ± 1.35
	Meaningfulness	4~28	19.13 ± 3.70	4.78 ± 0.93
Self-efficacy	Total score	10~40	24.55 ± 6.24	2.46 ± 0.62

### Correlation analysis of physical literacy with social support, sense of coherence, and self-efficacy in young and middle-aged patients with hypertension

The results revealed that the scores of physical literacy for young and middle-aged patients with hypertension were positively correlated with social support scores (*r* = 0.557, *P* < 0.01), sense of coherence scores (*r* = 0.392, *P* < 0.01), and self-efficacy scores (*r* = 0.466, *P* < 0.01). See Table 4 for details.

**Table 4:** Correlation analysis of physical literacy with social support, sense of coherence, and self-efficacy

	Mean (M)	Standard Deviation (SD)	Social support	Sense of coherence	Self-efficacy	Physical literacy
Social support	55.14	14.32	1			
Sense of coherence	59.27	13.60	0.427^**^	1		
Self-efficacy	24.55	6.24	0.502^**^	0.395^**^	1	
Physical literacy	62.30	13.92	0.557^**^	0.392^**^	0.466^**^	1

Note: ** indicates at 0.01 level (double tail), *P* < 0.01

### Structural equation model

Model Fit: The results showed that the χ2/df ratio was 2.647, which is less than 3. The RMSEA was 0.078, less than 0.08, and the GFI = 0.937, NFI = 0.950, RFI = 0.927, IFI = 0.968, TLI = 0.953, CFI = 0.968, all reaching the standard of 0.9. Therefore, the model fit indices were within acceptable ranges, indicating a good fit, and all path coefficients are statistically significant.

Mediation Analysis Using AMOS Software: Social support was the independent variable, with sense of coherence and self-efficacy as the mediating variables, and physical literacy as the dependent variable. Bootstrap testing was performed with 2000 resampling iterations, and a confidence level of 95% was selected for the bias-corrected confidence interval. If the confidence interval did not include 0, it indicated a significant mediation effect.

**Fig. 1:**
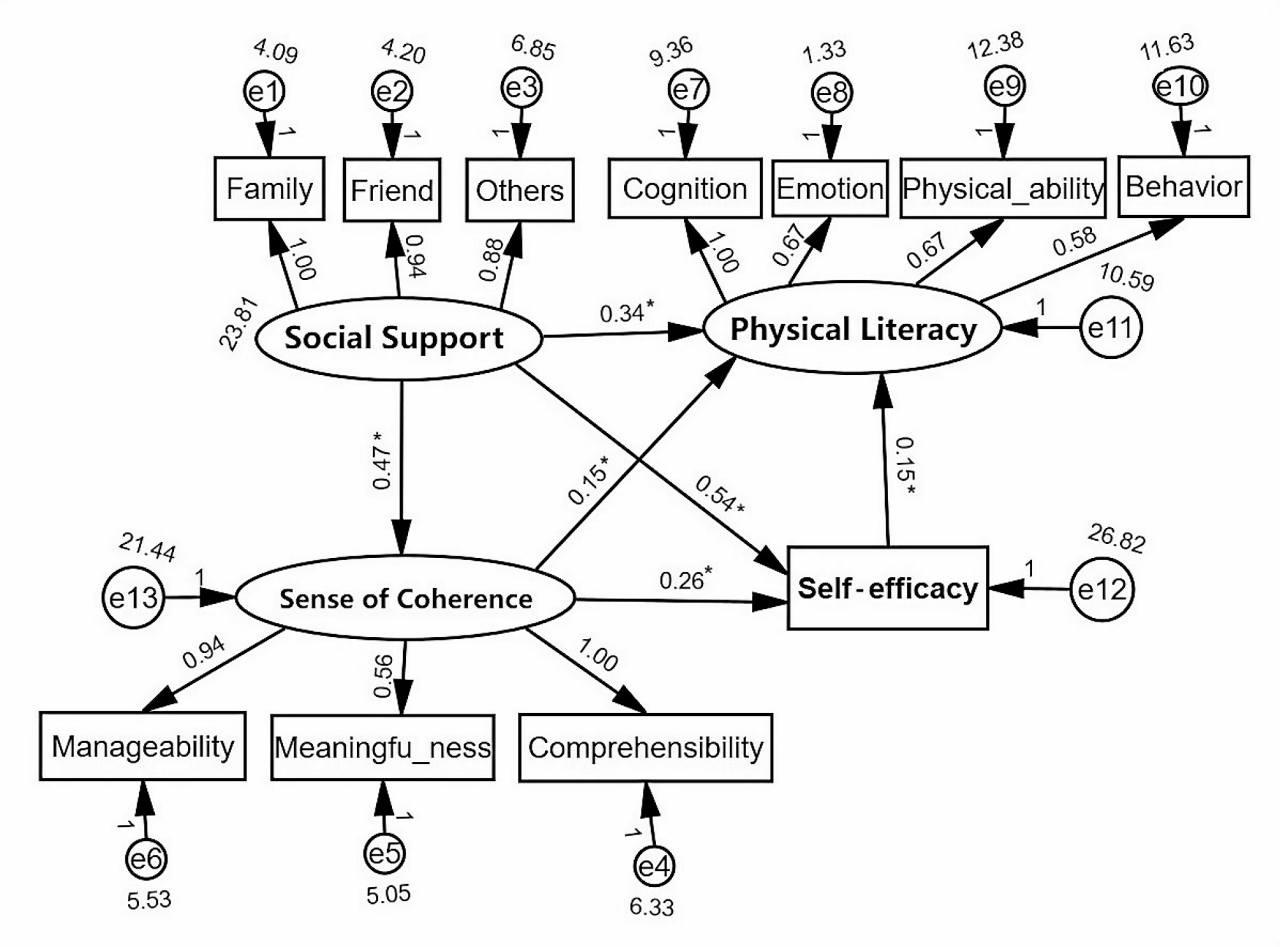
Structural equation model of physical literacy, social support, sense of coherence and self-efficacy Note: *:*P* < 0.05

The results showed that the total effect of social support on physical literacy is 0.505 (95% CI = 0.342–0.661, *P* < 0.05). The indirect effects were as follows: the coefficient of the path from social support to sense of coherence (path a) was 0.474 (95% CI = 0.308–0.658, *P* < 0.05); the coefficient of the path from sense of coherence to self-efficacy (path b) was 0.261 (95% CI = 0.090–0.433, *P* < 0.05); the coefficient of the path from self-efficacy to physical literacy (path c) was 0.151 (95% CI = 0.064–0.238, *P* < 0.05); the coefficient of the path from social support to self-efficacy (path e) was 0.542 (95% CI = 0.360–0.721, *P* < 0.05); and the coefficient of the path from sense of coherence to physical literacy (path f) was 0.147 (95% CI = 0.010–0.287, *P* < 0.05). The indirect effect of the model with social support → sense of coherence → self-efficacy → physical literacy (path a × path b × path c) was 0.019, with a 95% confidence interval of 0.005 to 0.047, indicating that the model of sense of coherence and self-efficacy as sequential mediators was valid. The indirect effect of the model with social support → sense of coherence → physical literacy (path a × path f) was 0.070, with a 95% confidence interval of 0.014 to 0.151, indicating the mediating effect of sense of coherence was valid. The indirect effect of the model with social support → self-efficacy → physical literacy (path e × path c) was 0.082, with a 95% confidence interval of 0.039 to 0.146, indicating the mediating effect of self-efficacy was valid. After adding the mediator variables sense of coherence and self-efficacy, the direct effect was 0.335 (95% CI = 0.166–0.507, *P* < 0.05), which remained statistically significant, indicating a partial mediation effect. In summary, the mediation effects of the three paths (social support → sense of coherence → physical literacy, social support → self-efficacy → physical literacy, and social support → sense of coherence → self-efficacy → physical literacy) were all significant, indicating multiple mediating effects of sense of coherence and self-efficacy between social support and physical literacy. Additionally, the total indirect effect of this study was 0.171, with the mediation effects of these three paths accounting for 40.94%, 47.95%, and 11.11% of the total indirect effect, respectively. See Table 5; Fig. 1 for detailed results.

**Table 5:** Path coefficients of structural equation model for physical literacy, social support, sense of coherence, and self-efficacy

Path	B	SE	95% CI	*P*
Social support→Sense of coherence(path a)	0.474	0.087	[0.308,0.658]	0.001
Sense of coherence→Self-efficacy(path b)	0.261	0.088	[0.090,0.433]	0.001
Self-efficacy→Physical literacy(path c)	0.151	0.044	[0.064,0.238]	0.001
Social support→Self-efficacy(path e)	0.542	0.093	[0.360,0.721]	0.001
Sense of coherence→Physical literacy(path f)	0.147	0.069	[0.010,0.287]	0.025
Social support→Physical literacy(total effect)	0.505	0.083	[0.342,0.661]	0.004
Social support→Physical literacy(direct effect d’)	0.335	0.086	[0.166,0.507]	0.001
Social support→Physical literacy(indirect effect)	0.170	0.043	[0.099,0.274]	0.000
Social support→Sense of coherence→Physical literacy(mediating effect 1)	0.070	0.034	[0.014,0.151]	0.016
Social support→Self-efficacy→Physical literacy(mediating effect 2)	0.082	0.027	[0.039,0.146]	0.000
Social support→Sense of coherence→Self-efficacy→Physical literacy(mediating effect 3)	0.019	0.010	[0.005,0.047]	0.000

Note: Estimated value B refers to standardized path coefficients

## Discussion

### The level of physical literacy, social support, sense of coherence, and self-efficacy among patients with hypertension needs improvement

The results indicated that the physical literacy of young and middle-aged patients with hypertension was at a moderate level, which aligned with the findings of Eighmy et al. [31]. This suggested improvement potential in cognition, emotion, physical competence, and physical activity behavior. Cognitively, this study confirmed that the recognition of the importance of physical activity in blood pressure control among these patients needed to be enhanced. Patients’ cognitive deficiencies may affect their physical activity behavior, resulting in suboptimal blood pressure management. Therefore, it is crucial to strengthen targeted health education to deepen patients’ understanding of hypertension and the benefits of physical activity, thereby guiding them to develop good physical activity habits, promoting blood pressure control, and cardiovascular health. Emotional enhancement is equally important. As noted by Aljama et al. [32]. , motivation and confidence are key factors influencing patients’ attitudes towards physical activity. This study also found that young and middle-aged patients with hypertension generally lacked motivation and confidence, which might have led to negative emotions during physical activity, affecting its continuity and effectiveness [33]. Hence, psychological support measures are needed to help patients build a positive emotional attitude, enhancing their intrinsic motivation and confidence in engaging in physical activities. Additionally, due to prolonged inactivity and poor lifestyle habits, the physical competence of these patients were generally weak. Therefore, it is necessary to develop appropriate physical activity training programs to gradually improve their physical competence and help them establish healthier lifestyles. In summary, there is a need to improve cognition, emotion, physical competence, and behavior among young and middle-aged patients with hypertension. Measures should be taken to enhance health education, psychological support, and physical competence training to improve their physical literacy levels.

Furthermore, the results of this study revealed that young and middle-aged patients with hypertension showed moderate levels of social support and self-efficacy, while sense of coherence was at a low level. Social support is crucial in disease management and psychological health, as it can provide emotional comfort and strengthen coping abilities. Consistent with existing research, these patients often faced insufficient social support, such as a lack of family and societal support [34]. Therefore, strategies to strengthen their social support networks and provide more emotional care and resource support are needed. The study also found low levels of sense of coherence and self-efficacy, possibly related to psychological stress and lack of confidence in physical activity. It is recommended to implement psychological interventions, such as cognitive-behavioral therapy, to help patients develop a positive mindset, enhance self-management abilities, and improve their sense of coherence and self-efficacy.

In conclusion, this study revealed deficiencies in the cognition, emotion, physical competence, and behavioral aspects of physical literacy among young and middle-aged patients with hypertension, along with challenges in social support, self-efficacy, and sense of coherence. To address these issues, targeted health education, psychological support, and physical activity training measures are recommended to improve physical literacy, strengthen social support networks, and enhance psychological adjustment abilities, thereby better controlling blood pressure and improving overall health.

### Correlations between physical literacy, social support, sense of coherence, and self-efficacy in young and middle-aged patients with hypertension

The results showed that the physical literacy of young and middle-aged patients with hypertension had a positive correlation with social support, sense of coherence, and self-efficacy. Firstly, social support was positively correlated with physical literacy of young and middle-aged patients with hypertension. This is consistent with the findings of Lee et al. [35]. , indicating that patients receive support and encouragement from family, friends and medical teams when improving physical literacy, which is conducive to them actively adopting different physical activity patterns to improve physical literacy. Therefore, strengthening the construction of social support system has positive significance for improving the physical literacy level of patients.

Secondly, there was a positive correlation between sense of coherence and physical literacy of young and middle-aged patients with hypertension. This is similar to Johansson et al. [36]. and may be associated with a higher sense of sense of coherence implying a greater sense of understandability, control and meaning, and a greater likelihood of positive physical activity behaviors leading to improved levels of physical literacy. Therefore, addressing psychological health issues and enhancing psychological interventions to improve patients’ sense of coherence is essential for boosting their physical literacy.

Lastly, self-efficacy was positively correlated with physical literacy in young and middle-aged patients with hypertension. Similar to the findings of Jianan [37], higher self-efficacy means patients have greater confidence in managing their disease and maintaining health, encouraging them to engage in physical activities and improve their physical literacy [38]. Enhancing patients’ self-efficacy and confidence in health management is thus necessary to improve their physical literacy.

To sum up, this study found that social support, sense of coherence, and self-efficacy were positively correlated with physical literacy in young and middle-aged patients with hypertension, highlighting the importance of these factors in enhancing physical literacy. In order to improve patients’ physical literacy, intervention of these related factors should be considered comprehensively.

### Analysis of the inherent mechanism model between physical literacy and social support, sense of coherence, and self-efficacy in young and middle-aged patients with hypertension

The results indicated that social support had a direct impact on the physical literacy of young and middle-aged patients with hypertension, consistent with Zhang et al. [39]. Specifically, social support may directly contribute to the improvement of physical literacy by providing emotional care and practical assistance to enhance the ability and confidence of patients to perform physical activities. This underscores the critical role of social support in enhancing patients’ physical health.

Additionally, social support indirectly improved physical literacy through self-efficacy, consistent with previous related studies [35]. This relationship might be attributed to the encouragement, resources, and emotional support provided by social support, which motivate patients to engage in health-promoting behaviors. Social support increases patients’ confidence in managing their blood pressure, turning their beliefs into actions, such as regular exercise and increased physical activity, thereby improving physical literacy.

Furthermore, the study found that social support indirectly influenced physical literacy through sense of coherence and self-efficacy, similar to Xinmin et al. [40]. In this mechanism, social support strengthened patients’ sense of coherence, increasing their comprehensibility, manageability, and meaningfulness when facing stress. A higher sense of coherence led to greater confidence in blood pressure control, fostering stronger intrinsic motivation and self-efficacy. Enhancing self-efficacy motivates patients to engage in proactive health management behaviors, such as improving disease-related knowledge, enhancing physical competence, and increasing physical activity, thereby improving physical literacy.

Finally, the study also revealed that sense of coherence influenced physical literacy through self-efficacy, consistent with Feng Haiyan et al. [41]. This suggests that when patients’ sense of coherence improves, they gain a clearer understanding of their condition, feel a stronger sense of control, and find greater meaning in life. Consequently, their self-efficacy is enhanced. This boost in self-efficacy increases their motivation and confidence to engage in physical activities, thereby promoting an improvement in their physical literacy.

In conclusion, this study deeply explored the relationships among social support, sense of coherence, self-efficacy, and physical literacy in young and middle-aged patients with hypertension, revealing the intrinsic mechanisms. The findings indicated that social support not only directly positively impacted physical literacy but also indirectly influenced it through sense of coherence and self-efficacy, highlighting the importance of psychological factors in forming health behaviors. Specifically, social support facilitates a stable sense of psychological coherence and a strong sense of self-efficacy, thereby further enhancing the physical literacy of young and middle-aged patients with hypertension.

## Limitations

This study provides valuable insights, but it is important to note several limitations. The cross-sectional design precludes conclusions about causality, and longitudinal studies are needed to establish the direction of relationships between variables. Convenience sampling may have introduced selection bias, limiting the generalizability of the findings. Self-reported measures are susceptible to response biases, which could affect the results’ accuracy. Confounding factors such as comorbidities and lifestyle habits were not fully examined and could influence the outcomes. Additionally, the focus on young and middle-aged hypertension patients limits the applicability of the results to other age groups or conditions. More diverse samples are necessary for broader applicability.

## Conclusion

In conclusion, this study underscores the pivotal role of social support in enhancing physical literacy among young and middle-aged patients with hypertension, with sense of coherence and self-efficacy as key mediators. Our research offers a novel perspective by examining the combined impact of social support, sense of coherence, and self-efficacy on physical literacy. This integrated approach provides valuable insights for developing holistic interventions aimed at improving health outcomes in this population. The primary innovation of this study lies in its exploration of the multifaceted relationships among these factors, which are often studied in isolation. By highlighting their synergistic effects, our study paves the way for more effective, multidimensional intervention strategies. Additionally, our findings have practical implications for enhancing social support systems. We recommend creating comprehensive networks that integrate online platforms with offline community activities, alongside tailored education and resources. These strategies can help patients develop a stronger sense of coherence and improve self-efficacy, leading to better physical literacy and overall health outcomes.

## Data Availability

All data generated or analysed during this study are included in this published article.
